# Blockchain 6G-Based Wireless Network Security Management with Optimization Using Machine Learning Techniques

**DOI:** 10.3390/s24186143

**Published:** 2024-09-23

**Authors:** Ponnusamy Chinnasamy, G. Charles Babu, Ramesh Kumar Ayyasamy, S. Amutha, Keshav Sinha, Allam Balaram

**Affiliations:** 1 Department of Computer Science and Engineering, School of Computing, Kalasalingam Academy of Research and Education, Srivilliputtur 626126, Tamil Nadu, India; 2 Department of Computer Science and Engineering, Gokaraju Rangaraju Institute of Engineering and Technology, Bachupally, Hyderabad 500090, Telangana, India; charles1624@grietcollege.com; 3 Faculty of Information and Communication Technology, Universiti Tunku Abdul Rahman (UTAR), Kampar 31900, Malaysia; 4 Department of Computer Science and Engineering, School of Computing, Vel Tech Rangarajan Dr. Sagunthala R&D Institute of Science and Technology, Chennai 600062, Tamil Nadu, India; dramuthas@veltech.edu.in; 5 School of Computer Science, UPES, Dehradun 248007, Uttarakhand, India; keshav.sinha@ddn.upes.ac.in; 6 Department of Computer Science and Engineering, MLR Institute of Technology, Hyderabad 500043, Telangana, India; drbalaramallam@gmail.com

**Keywords:** blockchain, 6G networks, wireless network, security management, machine learning

## Abstract

6G mobile network technology will set new standards to meet performance goals that are too ambitious for 5G networks to satisfy. The limitations of 5G networks have been apparent with the deployment of more and more 5G networks, which certainly encourages the investigation of 6G networks as the answer for the future. This research includes fundamental privacy and security issues related to 6G technology. Keeping an eye on real-time systems requires secure wireless sensor networks (WSNs). Denial of service (DoS) attacks mark a significant security vulnerability that WSNs face, and they can compromise the system as a whole. This research proposes a novel method in blockchain 6G-based wireless network security management and optimization using a machine learning model. In this research, the deployed 6G wireless sensor network security management is carried out using a blockchain user datagram transport protocol with reinforcement projection regression. Then, the network optimization is completed using artificial democratic cuckoo glowworm remora optimization. The simulation results have been based on various network parameters regarding throughput, energy efficiency, packet delivery ratio, end–end delay, and accuracy. In order to minimise network traffic, it also offers the capacity to determine the optimal node and path selection for data transmission. The proposed technique obtained 97% throughput, 95% energy efficiency, 96% accuracy, 50% end–end delay, and 94% packet delivery ratio.

## 1. Introduction

Due to its affordability, portability, and ease of implementation, wireless sensor networks (WSNs) are among the best solutions for a wide range of real-time applications. WSN monitoring involves gathering data, sending it to the base station for post-processing analysis, and monitoring areas of interest. Specific WSN systems require a large number of sensor nodes. The battery life and memory capacity of these wireless nodes are also constrained [1]. Therefore, the management method for these WSN nodes must be able to control how they interact with each other and the access point to get the most out of these WSNs. For instance, the Internet Engineering Task Force (IETF) established the 6LoWPAN and ZigBee protocols to allow management in wireless sensor networks (WSNs) and to provide standard transmission over IEEE 802.15.4. These protocols enable short transmission times and usage of IEEE 802.15.4 in the 2.4 GHz range by contemporary management systems. An example of a link between WSNs based on IP addresses on various tiers is provided by 6LoWPAN IPv6. Additionally, it maps the network topology using the 6LoWPAN Low Power and Loss Network (RPL) standard; it secures the WSN connection using the AES encryption technique [2].

Nonetheless, these networks’ dynamic topologies will impact network routing methods, delay, multi-layer architecture, coverage, Quality of Services (QoS), and fault detection. In order to address the nature of the contexts for which these embedded devices are intended, it is essential to reevaluate the management of WSNs through the creation of new protocols for their incorporation. The two most significant issues facing WSNs are security and energy usage, since they both have an adverse effect on the other. As a WSN’s security complexity increases, so does a node’s power consumption [3]. Given the challenging environments in which these sensors might operate, the requirement for both is one of the issues that recent research in this field attempts to address.

Furthermore, it is imperative to reassess the implementation of traditional security procedures, called Triangle, which are defined by Confidentiality, Integration, and Authentication (CIA). Key exchange, encryption, and encrypting data between two communication devices are examples of traditional approaches [4]. Due to elements including wireless medium, short transmission range, ad hoc deployment, hostile environments, and limited energy, security is the main problem in WSNs. We have two distinct methods in WSNs—prevention-based and detection-based—to secure these sensors. Prevention-based strategies are the first line of defence against security attacks in WSNs. Cryptography is the primary component of a prevention-based strategy, which takes more time and resources to process. As a result, this method is not recommended for WSNs. However, detection-based solutions would be more appropriate, as they use misuse/signature or anomaly detection and need less time and resources. They describe the collection of anomalous network behaviour from earlier [5]. Next, they search for assaults that the technique had previously defined. Because the signature-based detection technique only understands the behaviour of assaults previously specified, it cannot identify new attacks.

Conversely, the anomaly detection method builds a model for typical network events by learning the behaviour of the typical environment. Data or occurrences that differ from typical data or occurrences are called anomalies. Anomaly detection identifies the anomalies based on a predetermined set of typical data and occurrences. Therefore, this type of variance detection can identify unidentified attacks. Anomaly detection has a significant false alarm rate despite having a notably high detection rate. The IoT system, including WSN, can now upgrade and break through bottlenecks thanks to the introduction of artificial intelligence (AI) technology. AI technology is thriving, with new algorithms emerging due to constant data volume increases and significant processing power advancements. Evolving algorithms are one of the most popular among them. Differential evolution (DE) and genetic algorithms (GAs) are two algorithms that draw inspiration from natural evolutionary occurrences. Some algorithms, such as pigeon-inspired optimisation (PIO), grey wolf optimiser (GWO), particle swarm optimisation (PSO), and cat swarm optimisation (CSO), draw inspiration from biological group behaviours. Specific algorithms, such as the sine cosine algorithm (SCA), multi-verse optimiser (MVO), QUATRE, and others, are associated with mathematical principles or physical theory. Owing to its exceptional resilience and adaptability, evolutionary algorithms can efficiently solve numerous challenging issues in theoretical research and engineering technology domains. It can also accomplish optimisation tasks for many objective functions based on copious amounts of data and an effective algorithmic method [6].

### Research Contribution

- To suggest an innovative approach for machine learning-based blockchain-based 6G wireless network security management and optimisation;
- The blockchain user datagram transport protocol with reinforcement projection regression is used to maintain the security of the deployed 6G wireless sensor network;
- Artificial democratic cuckoo glowworm remora optimisation is used to optimise the network. The method additionally assesses the efficacy of feedback control methods and forecasts optimal results for their implementation in industrial WSNs, guaranteeing increased efficiency and an extended network lifetime. In order to guarantee a more energy-efficient solution, the method also makes it possible to investigate potential trade-offs between power consumption and communication performance.

## 2. Literature Survey

Over the last ten years, powerful machine learning techniques have been adopted by WSNs more and more intensively. Chinnasamy et al. [7]’s suggested framework aims to address issues concerning security and authorization in IoT network access control. The system aims to ensure security, authorization, and encryption for data sharing via IoT networks. This article introduces a unique system that provides a data-sharing framework integrating a blockchain-based access control system for IoT devices. In addition, a detailed analysis against the sustainable development via blockchain technology is provided in [8].

Fan et al. [9] provided a brief overview of machine learning methods used in WSNs for information processing and network performance enhancement. A related survey was conducted by Mahmood et al. [10] that included machine learning applications in wireless ad hoc networks. Three well-known machine learning algorithms—reinforcement learning, neural networks, and decision trees—were applied to all WSN communication layers [11]. On the other hand, specific surveys that address the use of machine learning for certain WSN difficulties have also been published. In order to enable appropriate actions to be taken, Shanmathi et al. [12], for example, addressed the development of practical outlier identification algorithms, some of which are based on machine learning principles. In the meantime, computational intelligence techniques can address problems in WSNs, such as routing, task scheduling, localisation, optimal deployment, and data fusion and aggregation [13]. In this context, computational intelligence refers to a subfield of machine learning that focuses on methods inspired by biology, including evolutionary algorithms, fuzzy systems, and neural networks [14]. An energy-efficient intrusion detection technique that identifies vulnerable areas in the network deployment region requires maintenance in IoT-based wireless sensor networks with 5G technology [15]. The weak areas are located and then fixed to produce the required level of barrier coverage. Specifically, their suggested approach concentrates solely on one-way coverage for scenarios involving one or more intruders.

Several studies asserted that the network lifetime may be increased by using their suggested methodology and techniques. In a different study, the effects of heterogeneous WSNs deployed in either a uniform or a Gaussian distribution scenario were examined and reported [16]. The effect of sensor density and sensor node sensing range on intrusion detection probability was investigated. They discovered that heterogeneous WSNs outperformed homogeneous WSNs regarding intrusion detection performance for a given sensing range and sensor node density.

Similarly, Satori [17] produced an analytical model that considers potential routes an invader could take to cross a border zone. To determine how long an invader would take to traverse a border area, they created a method that considers the border area’s features and infiltration routes. This study’s suggested model can identify an incursion when a person crosses a forbidden border. Vembu and Ramasamy [18] investigated IoT malware networks’ functioning using ML classification techniques such as RF, KNN, and NB. According to the researchers, the most dependable findings were produced using the KNN approach. In order to enable SVM training on IoT data, Surenther et al. [19] showed how privacy problems may be resolved. If a trustworthy third party was not involved, both transactions could be completed in a single cycle. Employing this alternate strategy suggests less complexity than applying the standard SVM method. Therefore, the effective use of ML technology may result in lower security costs. Anomaly detection can stop several negative things from happening, like packet analysis monitoring and DoS attacks [20]. By facilitating network access, easing traffic congestion, and finding errors, machine learning also helps determine physical layer techniques. If machine learning techniques are applied, WSNs may become more accurate and less prone to failure. These two domains have significantly increased in recent years [21]. This proposed method considers DV-Hop localisation techniques based on PSO. The radio irregularity model also showed how the proposed method may be applied in an anisotropic network. Sivakumar et al. [22] proposed an MLP-orientated localisation strategy for UN localisation in UAV-assisted WSNs. Due to its higher localisation accuracy and deployment efficiency, the MLP localisation approach was chosen for extensive simulation research. The valuable position of the sensors was evaluated by further minimisation of the localisation error. By using the WOA, the localisation error was decreased. Their results demonstrate that the presentation measurements of the suggested method performed better than the limitations’ inclusion and limitation error measurements of the previous study. This study concentrated on the memory, processing power, energy, and bandwidth limitations seen in WSN contexts. Debasis et al. [23] examined the requirements that algorithms must meet to function under a WSN, including energy conservation, minimal communication overhead, and robustness against the dynamic nature of network structure. For a sorting problem, Gururaj et al. [24] suggested lightweight algorithms utilising a single-class Quarter Sphere Support vector machine (QSSVM). They attained the same level of accuracy as QSSVM but with significantly less computing complexity. Zhang [25] used a hyper-ellipsoidal one-class SVM to method average data. Next, in an online distributed environment, outliers are found. They concluded that the system’s meagre false alarm rate is caused by updating a method built during the learning phase. Chinnasamy et al. [26] proposed AI-driven intrusion detection systems, methods for detecting False Data Injection (FDI) attacks, anomaly-based intrusion detection systems, and adaptive robust state estimators, ultimately presenting a new intrusion detection model, the AI-IBDS, which utilises the Grey Wolf Algorithm and Artificial Neural Network (GWAANN) for enhanced detection of network intrusions in energy systems. The training data were modelled using a spherical support vector machine in the class quarter [27]. SVM- and PCA-based approaches were suggested by Kamruzzaman [28] for the anomaly identification process. In order to accommodate non-stationary time series data, the initial algorithm in this study was SVM, with a small quantity of adjustment made to the RBF kernel, followed by a PCA-based method. Implementing the subspace tracking approach using Orthonormal Projection Approximation reduced the computational cost caused by Eigen Decomposition (ED) because sample points close to the sphere’s centre did not significantly contribute to the determination of the hyper-sphere, and Support Vector Data Description (SVDD) was changed to a lower training complexity.

## 3. Sixth-Generation Wireless Sensor Network Security Management

**Figure 1:**
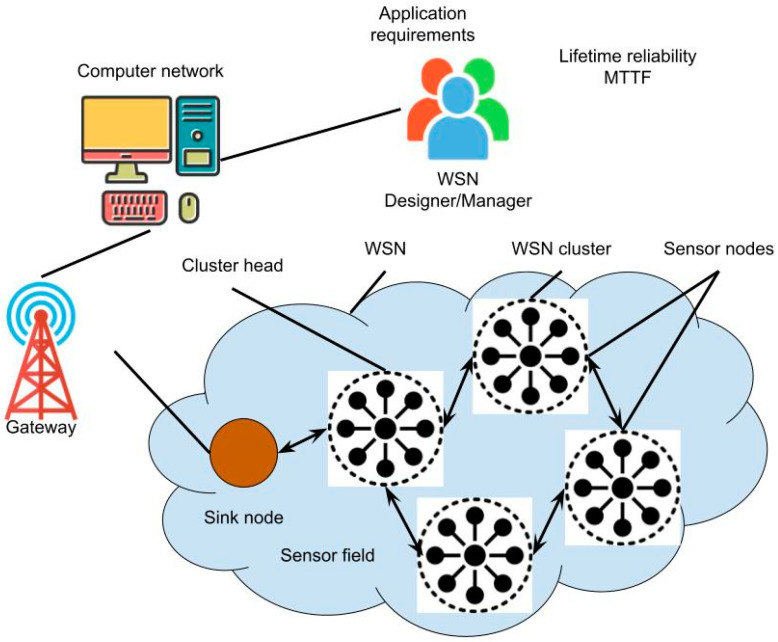
The proposed 6G wireless sensor network security analysis.

Our suggested system’s operation is shown in Figure 1. More precisely, a mobile network stationed on campus that utilises cutting-edge 6G technology and its advantages will link users to the Internet. Users may benefit from a faster and more responsive network experience thanks to the 6G network. Furthermore, they can send queries to cache servers based on the situation. The situation is as follows: if every user requests the same content item, only the initial request is processed; subsequent users will read the content item from the cache because it is cached there. A proxy cache server is required to mediate between the local server and the web servers, as seen in Figure 1. As a result, the proxy will receive the requests.

Reducing power consumption without compromising performance requires reliable communication channels and algorithm optimisation. WSNs are notoriously frugal with resources, particularly memory, processing speed, and network throughput. Safe routing is much more crucial because WSNs are vulnerable to a wide range of attacks. This paper proposes a novel secure routing technique for WSNs that can function safely in the presence of malicious nodes. Every node along the path has its status and trust value taken into consideration by the protocol. In order to determine the trust values of network nodes based on their performance levels, the suggested model considers a controller node (MN). The trust value represents the node’s chance of an attack based on past packet-forwarding behaviours. At the same time, status is a hybrid measure that includes the distance to the controller node and the residual energy.

Consequently, the protocol produces an optimal path regarding all available data and is safe from harmful attacks. The current WSN routing techniques need to balance security and energy efficiency. Trust-based routing protocols have a significant overhead because of the memory and processing power required to evaluate trust values, but they are resistant to various attacks. Since most existing routing algorithms are designed to minimise energy usage, they gradually extend the present path to establish a complete connection between any two nodes.

## 4. Proposed Blockchain User Datagram Transport Protocol with Reinforcement Projection Regression (UDTP-RPR)

Imagine a blockchain-enabled network topology representing IoT customers with two different node types, full-function nodes (FNs) and single-function nodes (SNs). In this architecture, SNs make up the majority of nodes and are enabled for transactions by the blockchain. The SNs’ low power and compact storage only allow them to send transaction information. Every SN may be in either an idle or active state. When an SN is transmitting data or is not in use, it is considered active. The following section describes the system model’s precise properties of their SN mode in the time domain. Nodes that support blockchain protocols are called FNs. In this network, FNs are responsible for building new blocks for the chain and verifying and storing the data sent from SNs. As a result, FNs must have powerful computers and lots of storage. FNs are connected over wired or high-speed wireless networks for security reasons.

With a density of λs and λf, respectively, all SNs and FNs are considered to be distributed as a homogeneous Poisson Point Process (PPP). There is a minimum distance, dmin, between the SNs and FNs for practical concern. Furthermore, we assume a distance dI for interference. Within dI, SNs can interfere with FNs at a distance. Then, SNs actively communicate data to FNs in the temporal domain. Since each packet’s length L is typically quite short (e.g., 1 KB in Bitcoin), the SNs’ active time t is very short, and t ≥ T holds. Consequently, the quantity of information packets received within a specific time frame is regarded as a Poisson distribution, with λaT as the parameter. Taking a circle with an FN centre and a dI radius as an example, this circle contains several SNs. Certain SNs are idle at any given time, while others are active and sending data to their N nearest FNs. If the N FNs receive the information, it is presumed that the transfer from the SN to the FN was successful. Route loss g ($D_{k1}$) affects signals received by FN k, where $D_{k1}$ is the separation between FN k and SN. Indicated by NI is how many SNs are active and within the dI of an FN by Equation (1):

(1) $${\mathrm{SINR}}_{k}\;\left(D_{k1},N_{R},D_{k2}\right)=\frac{Pg\left(D_{k1}\right)}{\sum_{i=1}^{N_{I}}\;Pg\left(D_{k2}^{\left(i\right)}\right)+\sigma }$$

It is necessary to assume that, during this time, all of the tributary host computers’ application layers have continued to produce payloads that have led to IP packets whose size is only constrained by the underlying Data Link layer’s payload size, which is typically the 1500 Bytes MTU imposed by the more than three-decades-old Ethernet. Furthermore, since there are no data to confirm or refute this basic hypothesis, one must rely on historical knowledge of the software ecosystem to draw the conclusion that there was not a significant shift in the kind and nature of users’ applications that would have caused the average packet size to increase by 66% in a short amount of time. Given the notion of distance, y1 is at a distance of 1, while y2 is at a distance of 2, since x2 and x3 are y2’s neighbours and are not decoded. After that, y4 and y5 are separated by a distance of 1, as y4 has an undecoded neighbour named x2, whereas y5 has x4.

When combined with a maximum likelihood decoder, we obtain an upper bound on the CDP decoder’s failure probability. With x being a row vector containing k data (input) bits and y denoting a vector of n encoding (output) bits received via a binary erasure channel (BEC), consider that every symbol is one bit without losing generality. Matrix H = [hi,j] is an adjacency matrix of size n × k, where an entry hi,j equals 1 if the j-th input symbol is next to the i-th encoding symbol. Then, the ML decoder is comparable to solving a HxT = y T system of linear equations. Encoding symbols with a distance of 0 or 1 at the CDP receiver results in an ACK message, which excludes the matching symbols from further transmissions. A realisation of matrix H where the recovery is of x2 is acknowledged in the first ACK message. Following that, H’s second column is set to zero. The adjacency matrix H not being of full rank is the same as ML decoder failure. Let pe represent the likelihood that the ML decoding rule will fail to decode an input bit j. Determine an upper bound on the ML decoder failure probability in the event that coding occurs without ACK messages by using the following information in Equation (2):

(2) $$p_{\varepsilon }=\mathrm{Pr}\;\left\{\exists x\in GF\left(2^{k}\right),x_{j}=1:Hx^{T}=0^{T}\right\}\leq \sum_{\begin{array}{c} x\in GP\left(2^{*}\right)\\ x,y=1 \end{array}}\;\mathrm{Pr}\;\left\{Hx^{T}=0^{T}\right\}$$

We give an upper bound using the same methods for coding with ACK messages. The UDTP (user datagram transport protocol) data transmission protocol exhibits notable distinctions from the other protocols. The system’s processed information regarding transferring an inactive file from the sender side is visible to the user. The system will describe the receiver’s and sender’s available buffer size in bytes. Furthermore, the bandwidth is expressed in megabits per second (Mbps). The Round-Trip Time (RTT), Time Stamp, and Mbps Send Rate are displayed excessively when the UDT protocol is used.

**Figure 2:**
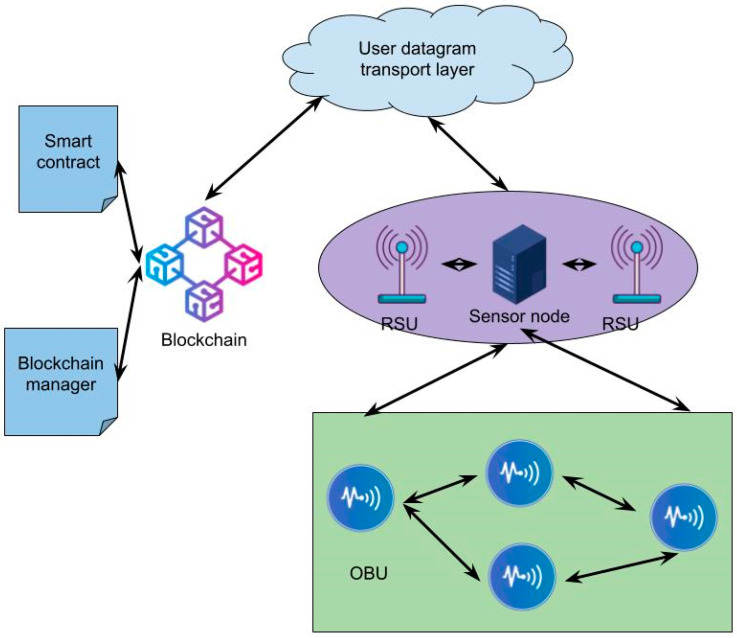
The architecture for user datagram transport protocol with blockchain analysis.

In contrast to the UDP, the UDT employs both negative and positive acknowledgement. Thus, using both acknowledgements contributes to the reliability of UDP-based transport protocols. Therefore, it also prevents packet loss. This information is helpful for the user. The architecture for the user datagram transport protocol with blockchain analysis is shown in Figure 2.

Maximising the predicted outcome through action learning in response to the environment is the focus of reinforcement learning (RL), a significant subfield of ML. Path planning optimisation learning is slowed down by the typical Q-learning algorithm’s low iteration efficiency and slow training speed because it is based on a decision method from Markov and needs prior information about the environment. The problem of maximising discounted reward can be rewritten as follows in Equation (3):

(3) $$\underset{\left((a1,\alpha \ldots ,as,k)\right)}{max}\;\underset{T\to \infty }{lim\;inf}\;E\left[\sum_{k=0}^{T-1}\;\sum_{i=1}^{N}\;-\delta^{k}tr\;P_{i,k}\right]$$

The action–value function is also known as the Q-factor. The projected future reward linked to acting on a while in state s is denoted by Q(s, a). For this issue, in Equation (4), the Bellman equation’s Q-factor variant is

(4) $$Q^{*}\left(s,a\right)=E\left[r+\delta \underset{\alpha^{\prime }}{max}\;Q^{*}\left(s^{\prime },a^{\prime }\right)|s,a\right]$$

where Q∗ (.,.) denotes the ideal Q-factor and s 0 denotes the value of the subsequent state given current state s and action a. By following action a ∗ (s) for every state s, we may determine a matching optimal stationary policy if we know Q∗ (.,.) using Equation (5):

(5) $$a^{*}\left(s\right)={\mathrm{argmax}}_{a}\;Q^{*}\left(s,a\right)$$

One can approximate Q∗ (s, a) for large MDPs by learning the weights associated with the function Q(s, a; θ) parameterised by a set of weights θ. In deep reinforcement learning, the function approximation Q(s, a; θ) is used. We employ two fully linked layers with outputs for every N!/(N − M)! = 120 action and two hidden layers, each with 1024 nodes. With δ = 0.95, the discount factor is set. The experience replay memory has a size of K = 20,000. After each iteration, the exploration parameter ε is attenuated from 1 to 0.01 at a rate of 0.999, meaning that ε ↑ max (0.999ε, 0.01). The ADAM optimiser, with an initial learning rate of e −4 and a learning rate degradation of 0.001, is utilised in neural network training. Every mini-batch has a size of 32. One update is made to the target Q-network every c = 100 time steps.

Given that this is the first RL method to ensure convergence to the optimal approach, it represents a significant advancement in the field. The literature provides evidence for its convergence. An equivalent definition of the Q-learning method in a particular Markov decision process (MDP) is given by Equation (6):

(6) $$Q_{t+1}\left(s,a\right)=\left\{\begin{array}{c} Q_{t}\left(s,a\right),\;\mathrm{if}\;s\neq s_{t}\;\mathrm{or}\;a\neq a_{t}\\ r\left(s,a\right)+\gamma \underset{d\in d}{max}\;Q_{t}\left(\delta \left(s,a\right),d^{\prime }\right),\;\mathrm{otherwise}\; \end{array}\right.$$

It is well known that the individual Q-learning method converges to the best Q(s, a) that maximises the reward. Through each iteration, Q-learning develops a better strategy p until it reaches the optimal strategy p. When two requirements are satisfied, Q-learning can ensure convergence, meaning that the method will converge arbitrarily quickly to the optimal strategy after an arbitrary amount of time: 1. As seen by Equation (7), the total of the squares must converge, but the sum of learning rates must necessarily diverge.

(7) $$\sum_{k=1}^{\infty }\;\;\alpha_{k}=\infty \;\mathrm{and}\;\sum_{k=1}^{\infty }\;\;\alpha_{k}^{2}<\infty$$

Every state–action must have infinite access, and a method with non-zero probability—that is, p(s, a)—must choose every action in every state.0 for every pair of state–action. Actually, this need can be satisfied by employing the E-greedy approach (where e.0). According to the earlier calculations, the value function Qt(St, At) of the well-known centralised control approach can converge to the optimal solution Q and increases monotonically with time t, much like in a single-agent environment. It has not been demonstrated that the model converges in a general sense in a multi-agent setting since it depends on the cooperative policies of other learning agents. Nonetheless, every CH and its CMs can communicate in the present cluster-routing environment since it is a particular MARL environment. As a result, the CMs can view the state of their CH node, but other CMs are unable to view the actions taken by CMs. The incentives that each agent earned are given back once their acts have been carried out in the environment. First, demonstrate that the value function Qt(St, At) of the centralised control mode, which is able to choose highest value function for intelligent individuals, is mapped to local action–value function qi t (St, ai t) of a single agent. When the Q-value is certain to increase, the method updates the Q-value, that is, for a state st and a joint action at = (a1 t, …, a t), for the agent i, by Equation (8).

(8) $$Q_{0}\left(s,a\right)=0\;\mathrm{for}\;\mathrm{all}\;s\in S,a\in A\;$$

This just makes predictions using a portion of the data and builds a local model. Every data point in the training set is converted into a weighting factor that indicates how much of an impact each data point has on the prediction process. A data point’s weighing factor indicates the degree to which the specific data point is pertinent to the current prediction. WSNs cannot use it since all training data must be stored in memory. Thus, we concentrated more on the gradual approach alone. The data matrix *Str*mXn is examined with ‘observations’ and ‘n’ variables that were gathered from the surroundings. After that, *Str*mXn is normalised to have a zero mean as well as unit variance. The normalised matrix is *Str*mXn. After determining the covariance matrix CV from the normalised matrix *Str*mXn, the *Str*mXn matrix is subjected to the Single-Value Decomposition procedure. Next, the principal component space as well as residual space become the two spaces into which the matrix mXn is projected. While the remaining components with less variation are held in the latter residual space, the former contains components that retain a majority of data variance, determined by Equation (9):

$$\hat{Str}=\hat{T}{\hat{P}}^{T}={\bar{Str}}_{m\times x_{n}}$$

$$\hat{Str}=\hat{T}{\hat{P}}^{T}={\bar{Str}}_{m\times x_{n}}\hat{P}{\hat{P}}^{T}={\hat{Str}}_{m\times X_{n}}\hat{CV}$$

(9) $$\tilde{Str}=\tilde{T}{\tilde{P}}^{T}={\tilde{Str}}_{m\times n}\tilde{P}{\tilde{P}}^{T}={\tilde{Str}}_{m\times n}\left(1-\tilde{CV}\right)$$

The loading matrix *P* is [$\hat{P}\tilde{P}$], where the set of the *l* and *n* − *l* Eigenvectors of the covariance matrix CV is contained in both $\hat{P}$ and $\tilde{P}$. After the kernel is constructed, the distances between each local model’s centre, *ck*, and each data point (*x*i, *yi*), which are inside the kernel, are used to compute the weights *w*ke,i. The following is how the Gaussian kernel is calculated by Equation (10):

(10) $$w_{k,1}=\exp\;\left(-\frac{1}{2}{\left(x_{i}-c_{k}\right)}^{T}D_{k}\left(x_{i}-c_{k}\right)\right)$$

The distance metrics denoted by *Dk* indicate the extent of the model’s validity zone. The term “receptive field” (RF) refers to the validity zone. Every receptive field has a different and optimised distance metric, *D*. Stochastic leave-one-out cross-validation can be used as an optimisation criterion. The RPR method’s prediction output, which is obtained by mixing locally weighted models, is defined in Equation (11):

(11) $$y_{\mathrm{pred}\;}=\frac{\sum_{k=1}^{N_{k}}\;w_{k}y_{k}}{\sum_{k=1}^{N_{k}}\;w_{k}}$$

where s is the anticipated input and *yk* = *βk* *s*. The local model k’s weight, Wk, is computed utilising k, which is the local model k’s receptive field (RF). The distance measure *D* and the regression parameter *β* (local), and the projection directions are the parameters that need to be learnt. *D* can be constructed as in Equation (12)

(12) $$D=\mathrm{hdiag}\;\left(\left[n_{1},n_{2},\ldots n_{n}\right]\right)$$

with the scaling parameter ‘ℎ’. In order to update local models later on, the learnt parameters are updated. Here, we demonstrated a dynamic threshold-generating technique within the detection process itself, as opposed to providing a threshold value as an input to the detection method. Because they could be influenced by physical factors like time and seasonal variations, the criteria under evaluation will not always remain the same. In the afternoon, the temperature might rise, but in the morning and evening, it might drop a little. To lower false rates, the threshold should therefore be dynamic. The dynamic threshold value is calculated using the sliding window idea. For the sliding window, forty samples were collected. So, the threshold value was evaluated from 40 past samples.

## 5. Network Optimisation Using Artificial Democratic Cuckoo Glowworm Remora Optimisation (ADCGRO)

Sout bees, observers, and hired bees make up the three groups within the artificial bee colony. We use the bee’s natural curiosity to find food sources first, then inform a bee passing by about what we have discovered. The spectator bee searches the area for a better food source, using the information passed down from the working bee. When the enhancement of the food supply surpasses a certain threshold, the observer bees quit the food source and turn into scout bees. Scout bees haphazardly search for a fresh food supply. Both hired and bystander bees carry out the exploitation in ABC, while scout bees oversee the exploration process, as described in Equation (13):

(13) $$v_{ij}=x_{ij}+\phi_{ij}\left(x_{ij}-x_{kj}\right)$$

where vi is new position of the i-th employed bee and xi is the previous one, whereas k is a random value between the number of bees used and 1, ϕij is a random number in the range of [−1, 1], and j is an arbitrary integer number between 1 and the issue dimension. Conversely, the observer bee evaluates every employed bee and chooses food based on the likelihood determined by Equation (14):

(14) $$p_{i}=\frac{fit_{i}}{\sum_{j=1}^{S\;V}\;fit_{j}}$$

fit is the fitness value of the employed bee’s answer, and Equation (15) is used to determine it.

(15) fit =11+fxi, fxi≥0 1+fxi, fxi<0

Similar to the employed bee, the observer bee updates its position and investigates the chosen food source using Equation (16). The definition of the objective function is the most crucial component that needs to be decided when using the Cuckoo algorithm for optimisation. Whether we want to optimise for system efficiency or system error minimisation depends on the objective function. The goal of this study is to minimise the inaccuracy in the system or the cost function. The following equations yield the Integral of Square Error (ISE), which is utilised to calculate cost function (fc):

$$e\left(t\right)=Pmax-P\left(t\right)$$

(16) $$ISE=\int_{0}^{\infty }\;e^{2}\left(t\right)dt$$

Similar to other optimisation methods, the cuckoo method starts with an initial population where every cuckoo has a specific spot in the problem space. Optimisation findings will be more accurate the more Cuckoos there are. Cuckoo population density causes the convergence rate to slow down. However, while parameter optimisation is carried out offline, convergence speed is unaffected. As a result, it is necessary to first ascertain the number of cuckoos that will deposit eggs. An array is used to introduce the problem variables’ values. We refer to these arrays as “habits” in the Cuckoo method. In the problem space, each habitat specifies a potential solution. It is necessary to have an array created by the values of the problem’s variables. The array termed “Habitat” in the cuckoo optimisation algorithm is utilised. In an optimisation problem, 1 * Nvar, which displays the cuckoo’s current position, indicates the habitat’s next position, or Nvar. Equation (17) defines this array.

(17) $$\mathrm{Nvarhabitat}\;=\;\left[x1,x2,x3,\ldots xNvar\right]$$

The variable’s values (x1, x2, x3, … x Nvar) each represent the number of floating points. The profit function f p of the current habitat is evaluated to determine its fitness (Equation (18)).

(18) $$\mathrm{profit}\;=f_{p}\left(\;\mathrm{habitat}\;\right)=f_{p}\left(x1,x2,x3,\ldots xNvar\right)$$

The cuckoo optimisation algorithm employs the maximum profit function, as was previously explained. In order to use this approach in the minimisation algorithm, the cost function must be multiplied by a negative sign. The natural glowworm phenomenon, in which a glowworm is lured to other glowworms that are brighter, served as inspiration for the GSO algorithm’s working mechanism. The GSO method is a swarm intelligence (SI) approach that mimics glowworm activity to optimise multimodal functions. Complex optimisation issues that are impossible or too difficult for the conventional approach to handle are resolved with the help of swarm intelligence algorithms. When employing SI, certain algorithms have a number of benefits and drawbacks. It is possible to spot glowworms while strolling through the meadows at night. These long-lasting, brightly flashing creatures brighten up the night. A luminous component known as luciferin is carried by every glowworm. As a result, every glowworm emits a light within a cave or at night. In a variable neighbourhood defined by a decision range and sensor range, each glowworm will communicate with the others. Due to their innate tendency to seek out brighter luciferin for both mating and prey, glowworms are always on the move within their neighbourhood. The luciferin update phase is impacted by the function value based on glowworm location. Each glowworm updates its luciferin level by adding the previous value to its luciferin quantity, which is based on how fit its present position is. A portion of the luciferin value is subtracted in order to simulate gradual deterioration of luciferin. Equation (19) is used to update glowworm luciferin, and the guidelines are provided by

(19) $$l_{i}\left(t+1\right)=\left(1-\rho \right)l_{i}\left(t\right)+yJ\left(x_{i}\left(x+1\right)\right)$$

where (xi(*t*)) is value of the objective function at agent *i*’s position at time *t*, *p* is the luciferin decay constant (0 < *p* < 1), *y* is the luciferin enhancement constant, and *li*(*t*) is the luciferin level associated with glowworm *i* at time *t*. Using the GSO idea as a guide, the glowworm or agent will seek out a higher luciferin level than its neighbour and move towards or around it in order to attract it. Furthermore, the glowworm will move towards its neighbour, who has a higher luciferin intensity, during this phase via the probabilistic method described by Equation (20):

(20) $$N_{1}\left(t\right)=\left\{j:d_{ij}\left(t\right)<r_{d}^{i}\left(t\right);l_{j}\left(t\right)<l_{j}\right\}$$

The collection of the neighbours of glowworm *i* at time *t* is given by Equation (20). A sensor range that is radially bounded (0 < *rd* *i* < *rs*) surrounds the variable. Equation (21) gives the probability of travel towards neighbour *j* ∈ *N*i(*t*) for each glowworm *i*.

(21) $$P_{ij}=\frac{l_{j}(t)-l_{i}(t)}{\sum_{k\in N_{i}(t)}\;l_{k}(t)-l_{i}(t)}$$

Equation (22) then represents the glowworm’s movement’s discrete-time model.

(22) $$x_{i}\left(t+1\right)=x_{i}\left(t\right)+s\left[\frac{x_{j}\left(t\right)-x_{i}\left(t\right)}{∥x_{j}\left(t\right)-x_{i}\left(t\right)∥}\right]\;$$

where || || is the Euclidean norm operator and *s* (>0) is the step size. The location of the glowworm *i* at time *t* in the m-dimensional real space *R* *m* is then represented by *xi* (*t*) ∈ *R* *m*. A neighbour range update phase utilised to identify several peaks in a multimodal function landscape is depicted in Equation (23). Next, let V0 be each glowworm’s initial neighbourhood range value. Each glowworm’s neighbourhood range update rule is adaptively updated using the following rule:

(23) $$r_{d}^{U}\left(t+1\right)=min\left\{r_{s},max\left\{0,r_{d}^{U}\left(t\right)+\beta \left(n_{t}-\left|N_{l}\left(t\right)\right|\right)\right\}\right\}\;$$

A GW (Gray Wolf) may vary if the separation between it and its closest neighbour is less than 4. Furthermore, a smaller step size could result in a slower coverage rate. Consequently, figuring out the ideal step size is really difficult. The step size in this inquiry will not be fixed; instead, it may change with each iteration depending on the GW. GW step size will be changed in multiple ways; a dynamic step size is expected to improve computational precision in the latter search space and speed up convergence in the earlier hunting stage. The following describes the remora optimisation algorithm (ROA) and how it works.

Free Travel: SFO Strategy—Equation (24) generates the elite notion of the algorithm, which was utilised for the method formulation of this method’s location update

(24) $$R_{i}^{t+1}=R_{\mathrm{best}\;}^{t}-\left(\;\mathrm{rand}\;\times \left(\frac{R_{\mathrm{best}\;}^{t}-R_{\mathrm{rand}\;}^{t}}{2}\right)-R_{\mathrm{rand}\;}^{t}\right)$$

where R t rand is a random location.

Experience Attack—Similar to how knowledge develops, periodically take little steps around the host to find whether or not it is necessary to change the host. The following formula in Equation (25) is utilised to model the aforementioned principles:

(25) $$R_{\mathrm{att}\;}=R_{i}^{t}-\left(R_{i}^{t}-R_{\mathrm{pre}\;}\right)\times \;\mathrm{randn}$$

where Ratt is a tentative step and Rpre is the prior iteration’s location, defined by Equation (26).

(26) $$f\left(R_{i}^{t}\right)>f\left(R_{\mathrm{att}\;}\right)$$

Equation (27) describes the updated position-updating formulas with modifications.

$$V_{i}\left(t+1\right)=D\times e^{a}\times \cos\;\left(2\pi a\right)+X_{\mathrm{best}\;}\left(t\right)$$

$$D=\left|X_{\mathrm{best}\;}(t)-X_{i}(t)\right|\;$$

$$a=\;\mathrm{rand}\;\times \left(b-1\right)+1$$

(27) $$b=-\left(1+\frac{t}{T}\right)$$

where D is the separation between the food and the remora. Additionally, the remora can create a minor step using the encircling prey mechanism in WOA, which is depicted as follows in Equation (28), to further enhance the quality of the solution:

$$X_{i}\left(t+1\right)=V_{i}\left(t+1\right)+A\times D^{\prime }$$

A=2×B× rand −B

$$B=2\times \left(1-\frac{t}{T}\right)$$

(28) $$D^{\prime }=V_{i}\left(t+1\right)-C\times X_{\mathrm{best}\;}\left(t\right)$$

where the i-th remora’s freshly created position is represented by Xi(t + 1). The remora factor, represented by the letter C in ROA, is set to 0.1. The remora is capable of locating its own food. In light of this, an innovative autonomous foraging mechanism is added to the fundamental remora optimisation algorithm to improve the remora’s search efficiency. With its more adaptable mode, the enhanced remora optimisation algorithm achieves a good equilibrium between exploration and exploitation. It is important to note that the computational complexity of the original algorithm will not grow with the use of this method. There is a certain universality to the suggested method because it also works with other optimisation algorithms.

## 6. Simulation Settings

Using network simulator-2, the suggested methodology is tested using between one hundred and two hundred sensors connected to different numbers of “gateways” spread throughout a 100 by 100 m^2^ area. The data points and the gates were initially assigned an energy of 100 joules and 200 joules, respectively. Here, we introduced a dynamic threshold-generating approach in the detection operation rather than providing a threshold value as the input to the detection method. The criteria being considered will only sometimes remain constant, as they may be influenced by physical factors like time and seasonal variations. Midday temperatures might rise, but they are lower early and late in the day. Thus, the threshold should be dynamic to lower the false rates. The dynamic threshold value is ascertained by applying the sliding window idea. Forty samples were collected for the sliding window. Thus, the last 40 samples are used to compute the threshold value. The same information is mentioned in Table 1.

## 7. Result Analysis Based on Wireless Network Security

There are 25 sensor nodes in total, each with a unique ID between 1 and 25. The nodes use an RTS/CTS control method to exchange data. Every 0.25 s, each node makes an attempt to send a packet with a probability of “P”. A collision occurs at the receiver when two nodes send data at the same time. The number of RTS packets a node receives in a minute is known as the request rate, or Rr. The average number of collisions that take place in a minute is known as the collision rate, or Rc. The values of these Rr and Rc parameters are averaged across 50 trial runs for every possible DoS attack likelihood, which varies from 0.1 to 1. After being normalised, these Rr and Rc values are shown in Table 2. The gauge of suspicion of an attack’s is called the probability of attack. It can be seen from Table 1 that Rc and Rr rise as the likelihood of an attack increases. NN-based approaches use the normalised values of these Rc and Rr parameters along with their related probabilities as training inputs.

**Figure 3:**
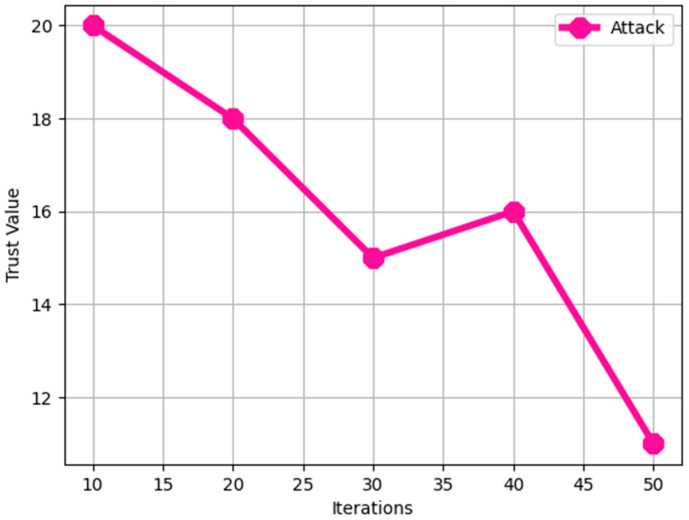
Trust value during a malicious node.

**Figure 4:**
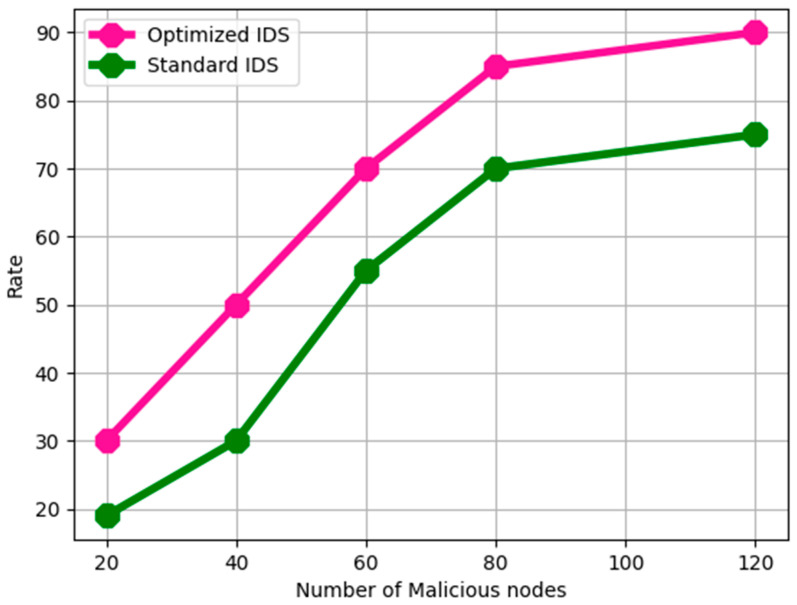
Rate of packet loss.

The number of nodes that can successfully communicate increases over time in the absence of malicious nodes. The degree of confidence in the case of an attack by a malicious node is shown in Figure 3 and Figure 4.

**Figure 5:**
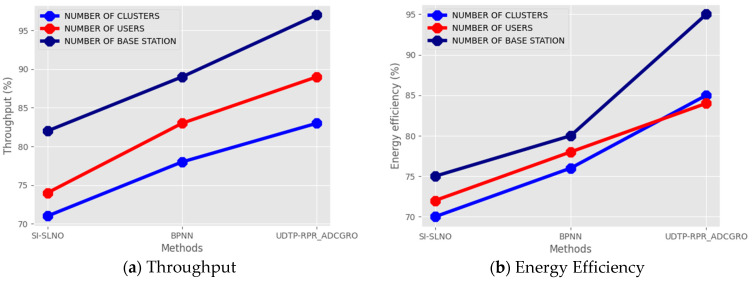

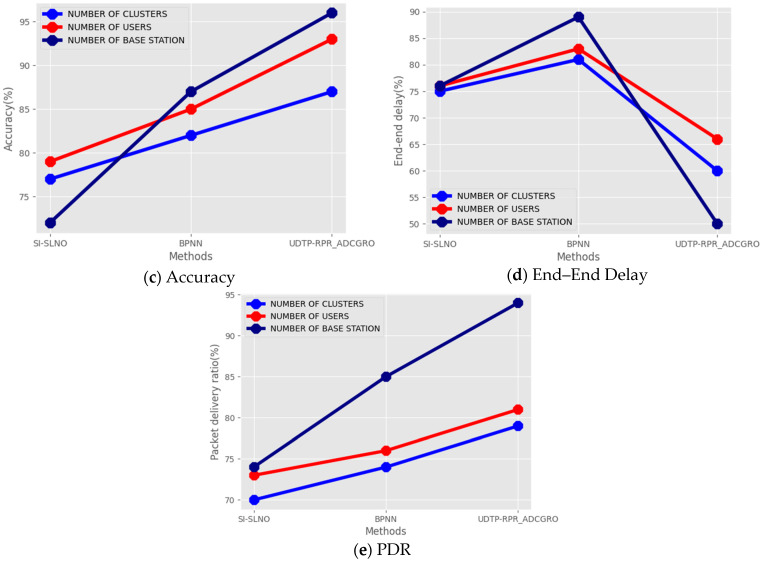
Comparison of multi-modal watermarked image datasets in terms of (**a**) throughput, (**b**) energy efficiency, (**c**) accuracy, (**d**) end–end delay, and (**e**) PDR.

Figure 4 illustrates the general trend in packet loss rates for all techniques, rising with the number of malicious nodes displaying aggressive behaviour. In order to execute Trusted Route Detection, only trusted nodes that are accessed are taken into account. This is achieved by combining MN node evaluation with the node trust factor and arbitrary node trust factor, and in a WSN, the trusted route aids in safe data transfer. The trusted Route Detection time levels for the suggested and present methods are displayed in Figure 5. Secure network architectures in WSNs prevent malicious attempts to manipulate the network’s routing infrastructure from interfering with route discovery.

Table 3 shows a comparison of various wireless sensor network parameters. Here, the proposed technique is analysed for the NUMBER OF CLUSTERS, NUMBER OF USERS, and NUMBER OF BASE STATIONS wireless sensor network parameters. The parametric analysis was carried out on throughput, energy efficiency, accuracy, end–end delay, and PDR.

Figure 5a–e show the comparative analysis of the multi-modal watermarked image dataset. Here, the proposed technique is throughput 83%, energy efficiency 85%, accuracy 87%, end–end delay 60%, and PDR 79%. While the existing SI-SLNO [18] attained a throughput of 71%, energy efficiency of 70%, accuracy of 77%, end–end delay of 75%, and PDR of 70%, the BPNN [19] attained a throughput of 78%, energy efficiency of 76%, accuracy of 82%, end–end delay of 81%, and PDR of 74% for the NUMBER OF CLUSTERS dataset. The proposed technique obtained 89% of throughput, 84% of energy efficiency, 93% of accuracy, 66% of end–end delay, and 81% of PDR. The existing SI-SLNO [18] attained a throughput of 74%, energy efficiency of 72%, accuracy of 79%, end–end delay of 76%, and PDR of 73%, and the BPNN [19] attained a throughput of 83%, energy efficiency of 78%, accuracy of 85%, end–end delay of 66%, and PDR of 81% for the NUMBER OF USERS dataset. For the NUMBER OF BASE STATIONS dataset, the proposed technique obtained 97% of throughput, 95% of energy efficiency, 96% of accuracy, 50% of end–end delay, and 94% of PDR. The existing SI-SLNO [18] attained a throughput of 82%, energy efficiency of 75%, accuracy of 72%, end–end delay of 76%, and PDR of 74%; the BPNN [19] attained a throughput of 89%, energy efficiency of 80%, accuracy of 87%, end–end delay of 89%, and PDR of 85%.

Attacks are categorised as ‘1’ for the probing category. Attacks against DoS are labelled with ‘2’, attacks against U2R with ‘3’, attacks against R2L with ‘4’, and attacks against the remaining average data with ‘5’. Forty-one features—basic, content, and traffic—are extracted from the various attacks and categorised into three groups. Thirty-three continuous and seven discrete features are the total acquired features classified as discrete and continuous. Comparing the suggested DL method to traditional ML methods, it is found that the latter achieves a lower detection accuracy. The suggested method identifies intrusion more accurately than the current methods because it optimises the use of hidden layers, deep characteristics, and appropriately selected features. Since the other three methods do not consider the classifier’s interaction with input, deleting certain superfluous internally dependent features is simple. Though the features alone offer substantial potential for discrimination, discrimination performance of those features is low when data containing these characteristics are analysed as a whole. The wrapper learning method weighs a possible subset’s advantages while considering prediction accuracy. Together, classifiers and feature selection make it possible to choose a subset of characteristics that will be helpful for learning.

## 8. Discussions

Some nodes were chosen randomly and given higher trust levels than others in this experiment. The proposed method successfully creates routes between very reliable nodes. The effectiveness of the recognition algorithm is evaluated using three metrics. The mean detection process for correctly identified malicious nodes, or reaction time, shows how fast malicious nodes can be found. The efficiency of our technique is reflected in the identification rate, which is calculated as the ratio of malicious nodes identified to all harmful nodes. In recent years, there has been a growing trend towards the optimisation of industrial wireless sensor networks’ sleep-state energy usage by the application of ML. In order to minimise energy consumption, as well as maximise performance, ML methods are trained to determine when it is most appropriate to enter and exit the sleep state. Industrial wireless sensor networks’ sleep-state energy consumption is customised to meet the demands of specific users and applications by utilising machine learning. By optimising the sleep-state energy usage with machine learning, variables like the application of kind, network traffic, sensor location, and time of day may be considered. Furthermore, industrial wireless sensor networks can increase lifespan by using machine learning to identify abnormalities and offer predictive maintenance solutions.

## 9. Conclusions

This study aims to present a unique approach for machine learning-based blockchain-based 6G wireless network security management and optimisation. The blockchain user datagram transport protocol with reinforcement projection regression is used in this study’s implemented 6G wireless sensor network security management. Subsequently, artificial democratic cuckoo glowworm remora optimisation is used for network optimisation. The DL method is employed in this research study as an IDS to improve the security of wireless sensor networks. The suggested deep learning-based method overcomes the drawbacks of the machine learning-based intrusion detection methodology. By employing cross-correlation to select the best characteristics, the suggested DNN’s computational complexity is decreased. The feature selection strategy aids in generalisation and guards against overfitting by lowering the model’s dimensionality and number of features.

On the other hand, it can help clarify how characteristics relate to associated values. To begin, test several machine learning techniques in a safe setting. This comprises an analysis of the model’s performance for every variation. After looking at all the measurements, it can be seen that the value for ε = 0.2 has increased the least when compared to ε = 0.4, 0.6, and 0.8. Minimal errors were obtained when ε = 0.4, near ε = 0.2. At ε = 0.8, there is a relatively significant inaccuracy. The routing model’s accuracy levels can be further improved by using optimisation models, and the parameters used to calculate trust can also be expanded for higher performance levels.

## Figures and Tables

**Table 1:** The simulation parameters of the proposed system.

Parameter/Operation	Details
Network Simulator Used	Network Simulator-2
Number of Sensors	100 to 200 sensors
Area	100 m × 100 m
Number of Gateways	Multiple gateways spread across the area
Initial Energy (Data Points)	100 joules
Initial Energy (Gateways)	200 joules
Threshold Generation Approach	Dynamic threshold-generating approach
Dynamic Threshold Calculation	Sliding window method
Sliding Window Size	40 samples
Threshold Calculation Method	The threshold value is computed using the last 40 samples
Purpose of Dynamic Threshold	To reduce false rates in detection

**Table 2:** Critical parameters averaged over 50 trial runs.

Probability of Attack	$R_{r}$	$R_{c}$
0.1	394.8	111.21
0.2	505.4	122.45
0.3	536.75	133.13
0.4	657.5	165.24
0.5	718.3	174.5
0.6	922.75	226.01
0.7	994.75	238.95
0.8	1058.49	261.38
0.9	1136.54	282.87
1	1195	303.65

**Table 3:** Comparison of various wireless sensor network parameters.

Network Parameters	Techniques	Throughput	Energy Efficiency	Packet Delivery Ratio	Accuracy	End–End Delay
NUMBER OF CLUSTERS	SI-SLNO [18]	71	70	70	77	75
	BPNN [19]	78	76	74	82	81
	UDTP-RPR_ADCGRO	83	85	79	87	60
NUMBER OF USERS	SI-SLNO [18]	74	72	73	79	76
	BPNN [19]	83	78	76	85	83
	UDTP-RPR_ADCGRO	89	84	81	93	66
NUMBER OF BASE STATIONS	SI-SLNO [18]	82	75	74	72	76
	BPNN [19]	89	80	85	87	89
	UDTP-RPR_ADCGRO	97	95	94	96	50

## Data Availability

The raw data supporting the conclusions of this article will be made available by the authors on request.
